# Teaching Digital Medicine in a Virtual Classroom: Impacts on Student Mindset and Competencies

**DOI:** 10.3390/ijerph20032029

**Published:** 2023-01-22

**Authors:** Julia Nitsche, Theresa S. Busse, Jan P. Ehlers

**Affiliations:** 1 Department of Didactics and Educational Research in Health Science, Faculty of Health, Witten/Herdecke University, 58448 Witten, Germany; 2 Institute of General Practice and Family Medicine (AM RUB), Medical Faculty, Ruhr University Bochum, 44801 Bochum, Germany

**Keywords:** education, digital medicine, technology-enhanced learning, digital competencies, technology, ehealth literacy, digital literacy

## Abstract

Digital competencies, as well as knowledge about digital medicine, are becoming increasingly relevant but are rarely reflected in teaching concepts at universities. One reason for this is probably the fact that they are not yet part of the curricula in many areas or countries (such as medicine in Germany). Therefore, courses that address digital competencies and intend to impart knowledge about digital medicine are not subject to any curricular specifications and have a correspondingly broad range of possible designs. This article reports findings from an investigation on an interdisciplinary and cross-faculty course on digital medicine. An online questionnaire was used to assess student attitudes toward digital medicine topics and conduct self-assessments of their digital competencies before and after the course. The aim of this study was to test whether such a course could influence students’ attitudes and competencies. Group comparisons revealed statistically significant changes. They proved that the described course and its content regarding digital transformation in healthcare and digital medicine had an impact on digital competencies and participant opinions on digital topics. In order to teach students important competencies for the 21st century, universities should offer more courses that address digital transformation and support students in improving their competencies.

## 1. Introduction

The future of medicine in digital societies raises great expectations and at the same time places high demands on people who work in the healthcare sector to define and establish themselves in a changing professional world [1,2].

In this context, change is often described by the term digitization, while this term in healthcare is defined and used inconsistently. Iyamu et al. [3] proposed a distinction between the subareas of modernization, digitization, and digital transformation, which formed the basis for this article. In this paper, modernization is understood as the technical process in which existing analog content is transformed into digital data. By extension, digitization encompasses the cultural and organizational changes necessary to incorporate and sustain technological advancements in service-delivery processes and achieve defined public health goals. In turn, digital transformation is seen as the complex and multifaceted process that fundamentally changes the culture, operating models, and goals of public healthcare, in which the focus is on the healthcare needs of the population.

On the one hand, opportunities for mobility and flexibility are changing; on the other hand, additional competencies are required. However, generational changes alone are not sufficient. Contrary to popular belief, younger generations are not automatically equipped with skills that help them to learn and work digitally [4]. For example, one study indicated that adolescents and young adults often acquired media skills in an unstructured way on their own, in part by learning through mistakes [5]. In addition, another study showed that although medical students had a positive view of digitization in the healthcare sector, they felt that they had little to no preparation for it during their studies [6]. These circumstances pose great challenges to existing structures such as teaching and learning methods [7]. While preparation for digital competencies is required [8], it is only partially addressed in the German National Competence-based Learning Objectives Catalog for Medicine (NKLM) 2.0 (https://nklm.de, accessed on 18 January 2023), which is not currently required to be implemented on a mandatory basis. The NKLM is a new basic development of the curricula for medical faculties in Germany that describes the competencies that are to be acquired by all medical students through the core curriculum. In addition to political bodies, experts from a wide range of disciplines, institutions, and the federal representation of medical students have been involved in its development [9]. The topicality and the simultaneous need for structured action was made clear in a review by Aulenkamp et al. that showed that 25 faculties in Germany were dealing with the topic of digital competencies in medical studies in an individual way even though there was no official curricular obligation to do so [10].

It is also crucial to clarify exactly what the term *digital competencies* encompasses. The decisive factor for the present study was an understanding of competence in the context of self-help guidance for coping with everyday professional problems. In this context, the terms digital literacy and eHealth literacy should be emphasized. Digital literacy means finding, understanding, and critically evaluating digital information [11], although there are wide ranges of terminologies, frameworks, and definitions of digital literacy. The European Union’s DigCom 2.2 framework provides a comprehensive overview of digital literacy [11]. By extension, eHealth literacy is a subcategory of digital literacy that refers to the ability to search for, find, understand, and critically evaluate health-related information within electronic media [12]. In this paper, the term digital competencies refers to digital literacy and eHealth literacy.

In addition, it should be emphasized that the development of skills for the use of digital technologies in the private sphere does not necessarily correspond to the evolution of competencies for digital technologies in professional settings such as healthcare [13]. Workers must be specifically trained for these professional competencies if they are to be relied upon during hectic day-to-day activities. This poses special challenges for universities in terms of training future healthcare personnel to become players in the digital medicine of the future by enabling them to participate in the digital world and achieving this as early as possible [14].

Above all, digital competencies in medicine are necessary to practice digital medicine. This includes, for example, the use of video consultations or apps. In this context, healthcare professionals are equally required to be users and knowledge mediators in order to guide patients and enable them to use digital medicine and develop their own digital competencies. For this, both knowledge about digital medicine and competencies in this area are important. This is critical to transforming paternalistic healthcare into care that is delivered at eye level by actively participating patients [15].

Even when digital competencies are trained in a technical and functional way, implicit teaching can also contribute to the formation of competencies. Active engagement in practical contexts seems to be particularly relevant [16]. In principle, digital courses can have an influence on attitudes toward digitization and the use of digital elements [17,18].

The interdisciplinary and cross-faculty course “Digital Medicine—How will data change the way we treat” at Witten/Herdecke University (UW/H), Germany, addresses the implicit teaching of digital competencies through the synchronous online format of the course and the explicit teaching of information regarding digital medicine. It is organized and delivered by the Chair of Didactics and Educational Research in Healthcare (Faculty of Health). In the sense of situated learning [19], the course attempts to place content on the topic of digital transformation in healthcare and digital medicine within situations and contexts, thereby integrating digital competencies into teaching. The main aim is to become familiar with future professional situations and digital realities in order to be able to evaluate them. Through their ability to evaluate, students are able to weigh up the use of digital technologies in their own professional environments and identify the advantages and disadvantages. Since the winter term (WT) of 2016/2017, the course has steadily gained participants and reached 300 students in the summer term (ST) of 2022 (total students at UW/H: 3,152). The particular challenge of this course lies in its delivery, which must be adapted to both the digital format and the large number of participants [20]. In comparison to our focus on attitudes and subcompetencies, student activities in this course were surveyed in another study that showed the successful use of co-moderation, discussion activities, group tasks, polls, and quizzes [21]. In addition, another challenge is that students from all semesters and majors can participate. This creates a very diverse range of prior knowledge, particularly regarding digital medicine and more generally regarding healthcare.

In accordance with its format and content, the course attempts to prepare students for the digital future of medicine. The focus is on imparting basic knowledge about digitization within the healthcare sector, particularly within the field of medicine, in order to teach students to assess the effects and consequences of digitization in their own professional fields. Thus, it serves the needs of students [22] and attempts to provide deeper insights into possible future challenges. The aim of this study was to verify whether the designed course actually had an impact on student attitudes toward the field of digital medicine and on digital competencies.

In this regard, surveying the successes achieved through university teaching posed challenges of potential bias due to student dependency ratios as well as the often large and diverse cohorts. Therefore, in our survey, the focus was on determining changes in the levels of knowledge and attitudes of students. In this context, the following research questions were investigated:

- What attitudes toward digitization and digital medicine do students bring to thecourse “Digital Medicine—How will data change the way we treat” at Witten/Herdecke University (UW/H)?
- Does the course have an impact on student attitudes toward digitization and digital medicine?
- Does the course have impacts on the students’ digital literacy, eHealth literacy, and digital medicine skills?

## 2. Materials and Methods

Ethical approval was obtained from the ethics committee of Witten/Herdecke University, Germany (approval code: 46/2020).

### 2.1. Intervention

The course was delivered digitally using the Adobe Connect meeting software (version 11.2) during the summer term of 2020 (ST20). The technical delivery did not run smoothly at the beginning due to the high server load, but it improved over the course of the semester. The course took place weekly on Thursdays from 18:00 to 19:30. The students received two credits in the European Credit Transfer and Accumulation System (ECTS) for their participation, which was tracked via login. Registration with real names was mandatory for this purpose. The digital delivery was planned before the outbreak of the COVID-19 pandemic; however, with the onset of the pandemic and the subsequent increase in digital teaching, the course gained attention because digital courses could be delivered without any restrictions. Some events were recorded with the permission of the speakers and uploaded to the YouTube account of the Chair of Didactics and Educational Research in Healthcare (https://www.youtube.com/@didaktikundbildungsforschu3503, accessed on 18 January 2023).

Each seminar was characterized by an impulse lecture followed by a subsequent discussion. The course lecturers (J.N., J.E., T.S.B., and a colleague from the Chair of Didactics and Educational Research in Healthcare) moderated the discussions and delivered some of the impulse lectures. However, other experts who gave impulse lectures on specific topics as guest lecturers also supported them. The lecturers also paid attention to the use of interactive elements in preparation for the sessions and provided support with applications for the participants and guest lecturers. Surveys and quizzes were used to engage the students, and they were also encouraged to participate in the discussions by agreeing or disagreeing [21]. The content of the course included current topics on the complexities of digital healthcare. In addition to the planned content, the course was overshadowed by the events of the COVID-19 pandemic. This additional unplanned content was selectively incorporated into topics repeatedly in order to discuss current affairs with the students (see the content overview in Table 1). In particular, digitization and its suddenly significantly increased relevance in the healthcare sector were incorporated.

### 2.2. Participants

As in previous semesters, students from the UW/H health and business and society faculties were able to participate in the course in ST20 [23]. The course took place within the framework of the Studium fundamentale (Stufu, fundamental studies) at UW/H. The goal of the Stufu program is for students to pursue and further their personal interests at an academic level outside of their core curriculum. Participation in the course required a basic level of digital literacy because both the registration for the course and the course itself were in digital form. Both employees from UW/H and external speakers design the Stufu program each semester, which includes the thematic areas of reflection space science, self and personality development, resource art, and critical contemporaneity. The special features of this program not only include dealing with content outside of subject-specific studies but also experiencing heterogeneous learning groups and profiting from interdisciplinarity [24].

### 2.3. Data Collection

A quasi-experimental evaluation design was chosen for the data collection. This design is typical for assessing the impacts of an intervention on certain outcome variables [25,26]. It is primarily used for program evaluation because key questions can be addressed on a limited scale [25]. Therefore, the questions were designed based on a previous qualitative data collection (see Section 2.4). This approach was particularly useful in this study because there was a concrete question with a directed starting point and it was not a matter of evaluating individual measures within the context of the intervention. In this study, the intervention was the course and the outcome variables were the student attitudes and competencies. This approach was chosen to allow us to focus on the influence of the intervention on the participating students.

In order to answer the research questions, a digital questionnaire was circulated during the course via the online survey tool Limesurvey (Version 3). This was a pseudonymous and nonexperimental cross-sectional survey (pre–post design). Participation in the questionnaire was voluntary. The research project was introduced during the first lecture of the course. Subsequently, the link to the pre-survey questionnaire (measurement time T1) was shared in the chat and sent by email to the participants of the course (on 23 April 2020). In addition, the link was also shared on Moodle, which was the learning platform for the course that all participants signed into. In the second lecture, participation in the questionnaire was referenced again and the link was shared in the chat. The post-survey questionnaire (measurement time T2) was sent out during the last lecture of the course via chat, email, and Moodle (on 9 July 2020).

### 2.4. Survey Design

The development of the nonvalidated content items was based on the previous content analysis of 16 reflection reports from previous semesters (winter term 2019/2020, summer term 2019, and winter term 2018/2019) by Kuckartz [27]. A classical approach was adopted in the context of medical education studies in which qualitative data were used to form hypotheses in order to test them using quantitative data [28]. Attention was also paid to what content students found particularly relevant and influential. The course items were supplemented with content that came up for the first time during the course.

The questionnaire was originally in German because the course was also conducted in German (Appendix A). Two people carried out the translation of the questionnaire independently. First, one person translated the questionnaire from German into English and then the second person, who had not seen the original questionnaire, translated the English version back into German. Both people then checked the German backtranslation together for discrepancies with the original questionnaire and to determine the final wording of the English questionnaire. The English version of the questionnaire can be found in Appendix B [29].

At the beginning of the questionnaire, participants had to create a pseudonym that consisted of the birth month of their mother (two digits), the last two letters of their father’s first name, and the first two letters of their mother’s first name. This offered participants the possibility to deny the use of their data after completing the questionnaire by requesting that their data not be used and providing their pseudonym (anonymously if needed). Additionally, this offered the chance to match data from the pre- and post-survey questionnaires. In the questionnaire, demographic data such as age, gender, and study program were required to be provided. Subsequently, 30 items had to be answered using a 6-point Likert scale (from 1 = I completely agree to 6 = I completely disagree). The items that were used were based on the content of the lecture. Statements were chosen that described (in the first person) the influence digitization had on the student’s life. The questionnaire ended with three items for the self-assessment of the digital subcompetencies of eHealth literacy, digital literacy, and digital medicine in German school grade format (from 1 = very good to 6 = insufficient).

It is important to note that this was purely an opinion survey and not a knowledge survey.

The pre–post design was chosen to evaluate the influence of the course on student attitudes toward the topics that were covered. For this purpose, each student’s knowledge level was assessed both before the intervention and after the intervention.

### 2.5. Data Analysis

After the data collection was completed, a statistical analysis was performed using SPSS version 29.0 with a focus on descriptive statistics. Group comparisons between the two measurement time points were calculated using the nonparametric sign test according to Dixon and Mood. This test was chosen because the Likert scales were ordinal scales and two dependent samples were compared pairwise [30]. Results were considered significant at a 5% significance level (*p* ≤ 0.050). For the additional testing of the results and to indicate the effect size, the Wilcoxon signed-rank test was also performed [31].

## 3. Results

Across ST20, an average of 257.73 (standard deviation 41.2) students participated in each lecture. Of the 346 students who applied for the course, 322 students attended at least one of the lectures. In total, 277 students received the certificate of attendance at the end of the course (i.e., they attended at least 80% of the lectures).

In total, 270 students completed the pre-survey questionnaire (T1) and 208 students completed the post-survey questionnaire (T2). After cleaning the data and removing duplicates and insufficiently completed questionnaires (dropout page 1), a total of 395 questionnaires remained from both measurement times. Using the number of students who completed the course with a certificate of attendance as the basis, the response rates of the questionnaires were 97.47% at T1 and 75.10% at T2.

The sample at T1 consisted of 235 records, of which 159 students (67.66%) assigned themselves to the female gender, 75 students (31.91%) assigned themselves to the male gender, and 1 person (0.43%) indicated a diverse gender assignment. The largest age group was 21–25 years old (N = 153; 65.11%). The age groups of 18–20 years (N = 31; 13.19%) and 26–30 years (N = 46; 19.57%) were represented to a similar extent, and 5 people (2.13%) were older than 30. In total, 11 students (4.68%) were assigned to the faculty of business and society (study programs management, philosophy, politics and economics, and cultural reflection), while 224 (95.32%) students were members of the faculty of health (study programs medicine, psychology, dentistry, and nursing science).

The sample at T2 consisted of 160 data records, of which 1 student (0.63%) indicated a diverse gender assignment, 53 students (33.13%) assigned themselves to the male gender, and 106 students (66.25%) assigned themselves to the female gender. Again, the 21–25-year-old group formed the largest proportion (N = 98; 61.25%), while the 18–20-year-old (N = 17; 10.63%) and 26–30-year-old (N = 40; 25%) groups were again represented in similar proportions, and 5 students (3.12%) were above the age of 30. In total, 10 students (6.25%) represented the faculty of business and society, while 150 students (93.75%) were from the faculty of health.

### 3.1. Student Attitudes

Overall, the students brought an open but critical attitude toward digitization in healthcare (particularly in medicine) to the course. The students tended to have fewer fears about changes in working environments due to digitization after the course in comparison to T1 and perceived digital transformation as beneficial and profitable, for example, with regard to the use of robots in working environments. Even though they believed that the amount of data to be processed would increase, their overall impression was that their work would not become significantly more demanding. They did not feel that digitization posed a threat to their jobs, but they did think that a rethink was required, especially with regard to ethical principles. Overall, there was a positive view of digitization in healthcare both before and after the course, with students seeing more advantages than disadvantages. However, this also entailed dealing with content thematically and comprehensively in order to be able to form opinions. A differentiated overview of all items is presented in Table 2. When comparing the faculties, the students from the faculty of business and society agreed more with 73.33% of the items on T1 than those from the faculty of health. The self-assessment of the subcompetencies at T1 differed positively for digital literacy and negatively for eHealth literacy and digital medicine. At T2, the students from the faculty of business and society agreed with 83.33% of the items more than those from the faculty of health did. The self-assessment of the subcompetencies at T2 differed positively for all three competency areas.

### 3.2. Student Competencies

The digital competencies of the students who attended the course were in the medium grade range. The evaluation involved the self-assessment of the three subcompetencies. The mean values at T1 and T2 showed improvements in all three competency areas after attending the course of just under 1 grade point (Table 3).

### 3.3. Influence of the Intervention

For the statistical testing of the influence of the intervention, 71 pseudonymized pairs at T1 and T2 were identified from the usable datasets. Within the framework of the Wilcoxon signed-rank test, there were significant changes in the results between T1 and T2 (Table 4).

The results indicated that the specific content covered in the sessions (i.e., apps, robots, telemedicine, etc.) produced changes in attitude. So, did the students believe that digital transformation brings more advantages than disadvantages? They seemed to be decisive that the content was taught to a high degree. Table 5 presents the results of the Wilcoxon signed-rank test. It was particularly interesting that the items “I have an idea how 3D printing works” and “I understand the benefits of virtual reality” also showed significant changes in attitude. According to the Wilcoxon signed-rank test, the item “For me, digital transformation in medicine brings more advantages than disadvantages” did not undergo a statically significant change in the directional test (alpha = 0.025). The effect sizes of the significant changes were in the medium (0.3–0.5) to strong (>0.5) range [32].

## 4. Discussion

This study addressed the effects of a digital medicine course on student attitudes, digital literacy, eHealth literacy, and knowledge of digital medicine. By means of a digital pseudonymized and nonexperimental cross-sectional survey (pre–post design), the initial situation and any changes after the course were surveyed. The intervention itself was a digital course that aimed to both implicitly and explicitly increase students’ skills regarding eHealth literacy and digital literacy as well as to impart knowledge about digital medicine.

The results showed that embedding content on digital medicine into the Stufu program at UW/H covered the current requirements for individual solutions [33]. Students need to deal with the topic of digital medicine and prepare themselves for their future careers by acquiring digital skills that include eHealth literacy and digital literacy. It is also essential for people outside of healthcare professions to be prepared for these challenges because patients and have both the skills for dealing with and knowledge about digital medicine. The integration of these complex topics is a current challenge facing the international university landscape (independently from the COVID-19 pandemic) [34]. Nevertheless, the pandemic situation has highlighted the importance of digital teaching and the challenges it involves [35]. Here, the focus is also on the continuing education of adult education staff, which is necessary to provide offerings such as the one described here and to ensure teaching even in pandemic or otherwise challenging situations.

Not all items in the questionnaire indicated a statistically significant change between T1 and T2. Nevertheless, it was clear from the results that the course had impacts on both student attitudes and digital competencies based on their self-assessments.

It was also clear that firmly embedding this content into the curriculum would be indispensable, meaningful, and purposeful [36]. The students themselves would ultimately be the ones to benefit from this and would be sufficiently prepared for the challenges in their later professional lives. This should be the goal of all university teaching.

In addition, we confirmed that although students have a basic understanding of digital content (such as the use of digital devices in private settings), these competencies do not automatically transfer to (pre-)professional situations [6,14,37,38]. Therefore, a differentiated consideration of digital competencies in professional environments is necessary. These demands also apply from the point of view of patients [39].

The course was able to change student attitudes toward individual subareas. The decisive factors were the positive development of digital subcompetencies and the feeling of being prepared for the consequences of digitization. The implicit teaching of digital competencies was also confirmed by the self-assessments [16]. This could lead to future opportunities to implement new technologies in the healthcare system in a targeted and meaningful way with trained specialists [40].

The competence and authenticity of teachers in terms of digital skills also seemed to be relevant [41,42], as did active engagement with the content, which was previously analyzed in an another study on this course [21]. In order to manage this situation, lecturers should be required to demonstrate their digital competencies.

The results also showed that the students who chose this course were those who had already dealt with the topic of digitization in healthcare in the past (whether by choosing this course format in past semesters or by some other means). The selection process at UW/H also takes into account current content such as digitization [43,44].

### Limitations

In future studies, more objective methods should be used to measure the increase in competence. These could include, for example, quizzes at the end of the course [21]. The limited number of participants at both measurement points was a limitation of our results. In subsequent pre-post studies on this course, a higher number of participants should be included in order to verify that the changes remain statistically significant.

It also would have been interesting to determine whether the course had already been attended by the students because this could have had an influence on the results at both T1 and T2. The students could have gained their prior knowledge from attending the course previously. The repetition of the course could also have led to the consolidation of knowledge about the content, although the content was differentiated in detail for each semester.

In addition, future surveys should track whether students also take advantage of other learning opportunities on the topics of digital literacy, eHealth literacy, and digital medicine outside of the course during the survey period in order to be able to control external influences as much as possible. The use of validated eHealth literacy test instruments such as the eHealth Literacy Scale (eHEALS) [45] could also allow future results to be interpreted more clearly. However, there is also a great deal of debate around these models because they have their own weaknesses and it is unclear which test is the most useful (depending on the target group and study goals).

Overall, our results highlighted the limitations of quantitative procedures in educational evaluation research. A critical reflection of the course by students was only possible to a limited extent and left room for interpretation. At this point, qualitative approaches need to be integrated in future research. A mixed-method approach could ideally combine the advantages of both types of approach. For this purpose, open-ended questions should be included in qualitative questionnaires so that the analysis of the answers is then qualitative. In addition, student evaluations could take place in digital rooms during the last course session that could be recorded anonymously (e.g., noting points from discussions on digital boards). In qualitative evaluation research, it is also important to guarantee the anonymity of participants because this is the only way to gain the most differentiated view possible without the influence of other dependencies. By using mixtures of methods, both formative and summative evaluations can be conducted.

## 5. Conclusions

Digital competencies and digitization in healthcare are no longer an idea but a reality for which students as patients and future healthcare professionals must be prepared. The particular challenge for universities is to find ways of supplementing curricula in meaningful ways because there is still no concrete integration of these topics and only ideas [42]. Students bring their own previous experiences to courses on digital medicine but in many areas, they are uncertain about the impact that digital medicine will have on their future professional environments. Implicit teaching through digital courses on digital medicine could address this challenge. We found that student competencies and attitudes toward this subject area could be influenced by our course. In terms of summative evaluation, these were the effects of the intervention. The active involvement of the participants also seemed to be crucial [21].

## Figures and Tables

**Table 1:** An overview of the “Digital Medicine—How will data change the way we treat” program in ST20.

Session	Topic	Content
1	Introduction to digital medicine ^1^	Introduction to the topic and a clarification of general conditions *
2	COVID-19 ^1^	Current scientific status and individual handling in the context of digital health *
3	Care robotics ^1^	The use of robots in care: a discussion of existing and desirable care scenarios *
4	E-learning ^1^	Digital learning using webinars and MOOCs and the professional use of social media *
5	Online psychotherapy ^2^	Background knowledge and examples of designs for online psychotherapy *
6	Apps in the medical sector ^1,2^	Background knowledge, examples and discussion of evidence for the effectiveness of apps in healthcare *
7	Telemedicine: Digital processes in medicine ^2^	The use of digital support in geriatrics: possibilities and experiences in telemedical care
8	Homo digitus ^2^	Changes in medical research and the activity of physicians due to digital transformation
9	Virtual reality in medicine ^2^	Treatment options for stroke and anxiety therapy within virtual reality
10	Digitization in pharmacies ^1^	The current digital status in pharmacies and possible further developments in Germany *
11	Site visit ^2,3^	Digital visit to the Bayer AG research center

^1^ Internal lecturer; ^2^ external lecturer; ^3^ elective date; * video recording available on YouTube.

**Table 2:** The mean values for the content items at T1 and T2 (6-point Likert scale: 1 = strongly agree; 6 = strongly disagree).

Item	Mean T1 (Two Decimal Places)	Mean T2 (Two Decimal Places)
I look forward to being supported by robots in my work.	3.13 (SD = 1.16; N = 216)	2.83 (SD = 0.96; N = 142)
I am afraid of changes in my daily work due to digitization.	4.24 (SD = 1.05; N = 216)	4.19 (SD = 1.12; N = 140)
For me, digital transformation in medicine brings more advantages than disadvantages.	2.61 (SD = 0.95; N = 207)	2.36 (SD = 0.75; N = 136)
I find the use of digital media in medicine profitable.	2.27 (SD = 0.86; N = 216)	2.15 (SD = 0.70; N = 143)
Digitization makes my work more demanding.	3.34 (SD = 1.06; N = 211)	3.33 (SD = 1.06; N = 141)
I can schedule my work more flexibly as a result of digitization.	2.88 (SD = 1.15; N = 208)	2.67 (SD = 1.12; N = 138)
The amount of information to be processed is increasing.	2.24 (SD = 1.03; N = 209)	2.18 (SD = 1.00; N = 138)
I feel well prepared for the consequences of digitization.	3.42 (SD = 1.21; N = 216)	2.14 (SD = 1.05; N = 141)
Digital education opportunities could help me in my job later on.	2.14 (SD = 0.89; N = 215)	2.00 (SD = 0.76; N = 139)
I have a generally positive attitude toward the expansion of digital healthcare offerings.	2.31 (SD = 0.85; N = 216)	2.27 (SD = 0.93; N = 139)
I see great potential for patient care with the digitization of medicine.	2.35 (SD = 0.82; N = 214)	2.18 (SD = 0.80; N = 140)
The use of artificial intelligence in medicine will replace my work.	4.34 (SD = 1.11; N = 209)	4.13 (SD = 1.21; N = 138)
Digitization means that ethical principles need to be revised.	2.14 (SD = 1.00; N = 208)	2.10 (SD = 0.97; N = 140)
Participatory work in medicine is increasing due to digitization.	2.98 (SD = 0.88; N = 170)	2.93 (SD = 0.91; N = 121)
Inter- and intraprofessional work in medicine is becoming easier due to digitization.	2.60 (SD = 0.99; N = 197)	2.47 (SD = 1.06; N = 133)
Digitization supports me in my everyday professional life.	2.55 (SD = 0.97; N = 210)	2.37 (SD = 0.93; N = 135)
For me, digital products in healthcare are an enrichment.	2.48 (SD = 0.97; N = 207)	2.48 (SD = 0.90; N = 134)
The use of products with artificial intelligence worries me.	3.37 (SD = 1.21; N = 208)	3.39 (SD = 1.11; N = 137)
I have a reflective opinion of digitization in healthcare.	2.88 (SD = 1.11; N = 203)	2.54 (SD = 0.98; N = 135)
In my opinion, Big Data is also an important topic in medicine.	2.04 (SD = 0.91; N = 182)	2.08 (SD = 0.84; N = 122)
I have an idea of how 3D printing works.	3.09 (SD = 1.49; N = 214)	2.59 (SD = 1.30; N = 140)
I understand the utility of virtual reality.	2.63 (SD = 1.17; N = 211)	2.39 (SD = 0.99; N = 139)
I use social networks for professional purposes.	3.89 (SD = 1.57; N = 211)	3.81 (SD = 1.55; N = 135)
In my opinion, the Digital Supply Act (Digitale Versorgung Gesetz, DVG) is crucial for digitization in healthcare.	2.65 (SD = 0.90; N = 157)	2.65 (SD = 1.03; N = 117)
I consider electronic patient records to be indispensable in today’s world.	2.05 (SD = 1.09; N = 212)	1.92 (SD = 1.12; N = 140)
Shared decision-making is an enrichment for all parties involved.	2.41 (SD = 0.93; N = 153)	1.92 (SD = 0.96; N = 116)
Telemedicine offers benefits for patients.	2.64 (SD = 1.03; N = 195)	2.47 (SD = 0.86; N = 135)
Precision medicine can be improved through digitization.	2.46 (SD = 0.89; N = 155)	2.50 (SD = 0.85; N = 117)
I am familiar with a large number of medical apps.	3.54 (SD = 1.38; N = 213)	2.88 (SD = 1.25; N = 139)
I consider the use of medical wearables to be unobjectionable.	3.59 (SD = 1.03; N = 156)	3.25 (SD = 0.97; N = 120)

SD = standard deviation

**Table 3:** The mean values for the self-assessment of the subcompetencies at T1 and T2 according to German school grade format (i.e., from 1 = very good to 6 = insufficient).

Item	Mean T1 (Two Decimal Places)	Mean T2 (Two Decimal Places)
My competencies in eHealth literacy are…	4.13 (SD = 1.32; N = 200)	3.17 (SD = 1.14; N = 128)
My competencies in digital literacy are…	3.98 (SD = 1.36; N = 198)	3.05 (SD = 1.10; N = 129)
My competencies in digital medicine are…	3.56 (SD = 1.31; N = 205)	2.67 (SD = 1.01; N = 131)

SD = standard deviation

**Table 4:** The results of the Wilcoxon signed-rank test on the pseudonymized pairs at T2.

Item	Z-Value	Assymp. Sig. (2-Sided)
I look forward to being supported by robots in my work.	−3.436	<0.001
For me, digital transformation in medicine brings more advantages than disadvantages.	−2.550	0.011
I feel well prepared for the consequences of digitization.	−2.623	0.009
Telemedicine offers benefits for patients.	−2.514	0.012
I am familiar with a large number of medical apps.	−4.484	<0.001
My competencies in eHealth literacy are…	−4.373	<0.001
My competencies in digital literacy are…	−4.287	<0.001
My competencies in digital medicine are…	−4.163	<0.001

**Table 5:** The results of the Wilcoxon signed-rank test on the pseudonymized pairs at T2.

Item	Z-Value	Assymp. Sig. (2-Sided)	Effect Strength (r)
I look forward to being supported by robots in my work.	−3.151 ^a^	0.002	0.4
I feel well prepared for the consequences of digitization.	−3.285 ^a^	0.001	0.42
I have an idea of how 3D printing works.	−2.585 ^a^	0.010	0.33
I understand the utility of virtual reality.	−2.351 ^a^	0.019	0.3
Telemedicine offers benefits for patients.	−2.474 ^a^	0.013	0.34
I am familiar with a large number of medical apps.	−4.609 ^a^	<0.001	0.6
My competencies in eHealth literacy are…	−4.694 ^a^	<0.001	0.66
My competencies in digital literacy are…	−4.250 ^a^	<0.001	0.6
My competencies in digital medicine are…	−4.251 ^a^	<0.001	0.58

^a^ Based on positive ranks.

## Data Availability

The datasets from this study are available from the corresponding author upon reasonable request.

## Appendix A

**Table A1:** Online questionnaire (original German version).

Pseudonym		
Geburtsmonat der Mutter (Zweistellig)	Letzten 2 Buchstaben Vorname des Vaters	Ersten 2 Buchstaben Vorname der Mutter
**Demographische Daten**		
Datum:		
		Bitte zutreffendes ankreuzen
Geschlecht	Weiblich	
	Männlich	
	Divers	
Alter	Unter 18 Jahre	
	18–20 Jahre	
	21–25 Jahre	
	26–30 Jahre	
	31–35 Jahre	
	36–40 Jahre	
	41–45 Jahre	
	45–50 Jahre	
	Über 51 Jahre	
Department	Medizin Vorklinik	
	Medizin Klinik	
	Zahnmedizin Vorklinik	
	Zahnmedizin Klinik	
	Psychologie Bachelor	
	Psychologie Master	
	Pflegewissenschaften	
	PPÖ Bachelor	
	PPÖ Master	
	Management Bachelor	
	Management Master	
	KuRe Bachelor	
	KuRe Master	

**Digitalisierung im Gesundheitswesen.**

Im Folgenden sind einige Aussagen zur Digitalisierung im Gesundheitswesen aufgelistet. Bitte kreuze an, in welchem Maße Du der Aussage zustimmst.

	**Ich Stimme…Zu**					
	**Voll und Ganz**	**Größtenteils**	**Eher**	**Eher Nicht**	**Größtenteils Nicht**	**Gar Nicht**
Ich freue mich darauf, in meiner Arbeit durch Roboter unterstützt zu werden.						
Ich habe Angst vor der Veränderung meiner täglichen Arbeit durch Digitalisierung.						
Der digitale Wandel in der Medizin bringt für mich mehr Vorteile als Nachteile.						
Ich finde den Einsatz von digitalen Medien in der Medizin gewinnbringend.						
Durch die Digitalisierung wird meine Arbeit anspruchsvoller.						
Ich kann meine Arbeit durch die Digitalisierung flexibler einteilen.						
Die zu verarbeitende Informationsmenge nimmt immer mehr zu.						
Ich fühle mich gut auf die Folgen der Digitalisierung vorbereitet.						
Digitale Bildungsmöglichkeiten können mir in meinem Beruf später helfen.						
Ich stehe dem Ausbau digitaler Gesundheitsangebote grundsätzlich positiv gegenüber.						
In der Digitalisierung der Medizin sehe ich für die Patientenversorgung große Potentiale.						
Durch den Einsatz von Künstlicher Intelligenz in der Medizin wird meine Arbeit ersetzt.						
Durch die Digitalisierung müssen ethische Grundsätze überarbeitet werden.						
Die partizipative Arbeit in der Medizin nimmt durch die Digitalisierung zu.						
Die inter- und intraprofessionelle Arbeit in der Medizin wird durch die Digitalisierung leichter.						
Die Digitalisierung unterstützt mich in meinem beruflichen Alltag.						
Digitale Produkte im Gesundheitswesen sind für mich persönlich eine Bereicherung.						
Die Nutzung von Produkten mit Künstlicher Intelligenz bereitet mir Sorge.						
Ich habe eine reflektierte Meinung zur Digitalisierung im Gesundheitswesen.						
Big Data ist meiner Meinung nach auch in der Medizin ein wichtiges Thema.						
Ich habe eine Vorstellung wie 3D Druck funktioniert.						
Ich verstehe den Nutzen von Virtual Reality.						
Soziale Netzwerke nutze ich auch für berufliche Zwecke.						
Das Digitale Versorgung Gesetz (DVG) ist meiner Meinung nach entscheidend für Digitalisierung im Gesundheitswesen.						
Eine elektronische Patientenakte halte ich in der heutigen Zeit für unumgänglich.						
Shared Decision Making ist für alle Beteiligten eine Bereicherung.						
Telemedizin stellt einen Mehrwert für Patient*innen dar.						
Precision Medicine kann durch die Digitalisierung verbessert werden.						
Ich kenne eine Vielzahl von medizinischen Apps.						
Den Einsatz von Medizin-Wearables halte ich für unbedenklich.						

Im Folgenden sind Bereiche Digitaler Kompetenzen aufgelistet. Bitte kreuze an, mit welcher Note Du Deine Kompetenzen in dem jeweiligen Bereich einschätzt.

	**1** **Sehr Gut.**	**2** **Gut.**	**3** **Befriedigen.**	**4** **Ausreichend.**	**5** **Mangelhaft.**	**6** **Ungenügend.**
Meine Kompetenzen in eHealth Literacy sind …						
Meine Kompetenzen in Digital Literacy sind …						
Meine Kompetenzen in Digital Medicine sind …						

## Appendix B

**Table B1:** Online questionnaire (translated English version).

Pseudonym		
Month of Birth of the Mother (Two Digits)	Last 2 Letters First Name of Father	First 2 Letters First Name of Mother
Demographic data		
Date:		
		Please mark with a cross
Gender	Female	
	Male	
	Divers	
Age	under 18 Years	
	18–20 Years	
	21–25 Years	
	26–30 Years	
	31–35 Years	
	36–40 Years	
	41–45 Years	
	45–50 Years	
	over 51 Years	
Department	Medicine Preclinic	
	Medicine Clinic	
	Dentistry Preclinic	
	Dentistry Clinic	
	Psychology Bachelor	
	Psychology Master	
	Nursing Sciences	
	PPÖ Bachelor	
	PPÖ Master	
	Management Bachelor	
	Management Master	
	KuRe Bachelor	
	KuRe Master	

**Digitization in the healthcare sector.**

Some statements on digitization in the healthcare sector are listed below. Please mark the extent to which you agree with the statement.

	**I**					
	**Agree Very Strongly**	**Agree Strongly**	**Agree**	**Disagree**	**Disagree Strongly**	**Disagree Very Strongly**
I look forward to being supported by robots in my work.						
I am afraid of changes in my daily work due to digitization.						
For me, digital transformation in medicine brings more advantages than disadvantages.						
I find the use of digital media in medicine profitable.						
Digitization makes my work more demanding.						
I can schedule my work more flexibly as a result of digitization.						
The amount of information to be processed is increasing.						
I feel well prepared for the consequences of digitization.						
Digital education opportunities could help me in my job later on.						
I have a generally positive attitude toward the expansion of digital healthcare offerings.						
I see great potential for patient care with the digitization of medicine.						
The use of artificial intelligence in medicine will replace my work.						
Digitization means that ethical principles need to be revised.						
Participatory work in medicine is increasing due to digitization.						
Inter- and intraprofessional work in medicine is becoming easier due to digitization.						
Digitization supports me in my everyday professional life.						
For me, digital products in healthcare are an enrichment.						
The use of products with artificial intelligence worries me.						
I have a reflective opinion of digitization in healthcare.						
In my opinion, Big Data is also an important topic in medicine.						
I have an idea of how 3D printing works.						
I understand the utility of virtual reality.						
I use social networks for professional purposes.						
In my opinion, the Digital Supply Act (Digitale Versorgung Gesetz, DVG) is crucial for digitization in healthcare.						
I consider electronic patient records to be indispensable in today’s world.						
Shared decision-making is an enrichment for all parties involved.						
Telemedicine offers benefits for patients.						
Precision medicine can be improved through digitization.						
I am familiar with a large number of medical apps.						
I consider the use of medical wearables to be unobjectionable.						

The areas of digital competencies are listed below. Please mark with a cross the grade with which you assess your competencies in the respective area.

	**1** **Very Good**	**2** **Good.**	**3** **Satisfactory.**	**4** **Sufficient.**	**5** **Poor.**	**6** **Insufficient.**
My competencies in eHealth literacy are						
My competencies in digital literacy are						
My competencies in digital medicine are						
